# Impact of motivational feedback on levels of physical activity and quality of life by activity monitoring following knee arthroplasty surgery—protocol for a randomized controlled trial nested in a prospective cohort (Knee-Activity)

**DOI:** 10.1186/s12891-024-07878-0

**Published:** 2024-10-02

**Authors:** Cecilie Dollerup Skov, Martin Lindberg-Larsen, Uffe Kock Wiil, Claus Varnum, Hagen Schmal, Charlotte Myhre Jensen, Anders Holsgaard-Larsen

**Affiliations:** 1 Department of Orthopedic Surgery and Traumatology, Odense University Hospital, Odense C, Denmark; 2 Department of Clinical Research, University of Southern Denmark, Odense C, Denmark; 3 SDU Health Informatics and Technology, The Maersk Mc-Kinney Moller Institute, University of Southern Denmark, Odense C, Denmark; 4 Department of Orthopedic Surgery, Lillebaelt Hospital Vejle, Vejle, Denmark; 5 Department of Orthopedic and Trauma Surgery, University Medical Center, Faculty of Medicine, University of Freiburg, Freiburg, Germany; 6 Department of Orthopedics and Traumatology, Department of Clinical Research, Odense University Hospital, & Orthopedic Research Unit, University of Southern Denmark, Odense C, Denmark

**Keywords:** Knee arthroplasty surgery, Knee osteoarthritis, Motivational feedback, App, RCT

## Abstract

**Background:**

Evidence on how to improve daily physical activity (PA) levels following total knee arthroplasty (TKA) or medial uni-compartmental knee arthroplasty (mUKA) by motivational feedback is lacking. Moreover, it is unknown whether a focus on increased PA after discharge from the hospital improves rehabilitation, physical function, and quality of life. The aim of this randomized controlled trial (RCT) nested in a prospective cohort is (a) to investigate whether PA, physical function, and quality of life following knee replacement can be increased using an activity monitoring device including motivational feedback via a patient app in comparison with activity monitoring without feedback (care-as-usual), and (b) to investigate the potential predictive value of PA level prior to knee replacement for the length of stay, return to work, and quality of life.

**Methods:**

The study is designed as a multicenter, parallel-group, superiority RCT with balanced randomization (1:1) and blinded outcome assessments. One hundred and fifty patients scheduled for knee replacement (TKA or mUKA) will be recruited through Odense University Hospital, Denmark, Vejle Hospital, Denmark and Herlev/Gentofte Sygehus, Denmark. Patients will be randomized to either 12 weeks of activity monitoring and motivational feedback via a patient app by gamification or 'care-as-usual,' including activity monitoring without motivational feedback. The primary outcome is the between-group change score from baseline to 12-week follow-up of cumulative daily accelerometer counts, which is a valid proxy for average objectively assessed daily PA.

**Discussion:**

Improving PA through motivational feedback following knee replacement surgery might improve post-surgical function, health-related quality of life, and participation in everyday life.

**Trial registration:**

ClinicalTrials.gov, ID: NCT06005623. Registered on 2023–08-22.

**Trial status:**

Recruiting.

**Supplementary Information:**

The online version contains supplementary material available at 10.1186/s12891-024-07878-0.

## Background

### Burden of disease

Musculoskeletal disorders, including knee osteoarthritis (OA), result in decreased physical activity (PA), leading to an increased incidence of chronic diseases [1]. Moreover, knee OA leads to work-related inactivity, a vicious downward spiral of increased symptoms, and a marked decrease in quality of life [2, 3]. The lifetime risk of developing symptomatic knee OA is estimated to be 45% [4].

### Total- or uni-compartmental knee arthroplasty and patient satisfaction

Knee replacement surgery by total knee arthroplasty (TKA) and medial uni-compartmental knee arthroplasty (mUKA) are two of the most frequently performed procedures in orthopedic surgery [5], and the lifetime risk of undergoing knee replacement if one suffers from OA is 30% [6]. Worldwide, over a million patients receive knee replacement surgery each year, and the numbers are expected to increase further due to the growing elderly population [7].

Despite successful surgical procedures, knee replacement patients still demonstrate decreased function and PA level, earlier retirement, less income, increased cost for health and home care compared to matched controls [8, 9]. Moreover, nationwide data underline the need for optimized treatment and/or rehabilitation as 20% of patients are dissatisfied one-year post-surgery, and poor postoperative knee function is negatively associated with patient satisfaction [10].

### Physical activity

Early mobilization on the day of surgery and a short length of hospital stay (median one day) have been achieved using the so-called fast-track protocols for knee replacement [11, 12]. However, little is known about the actual PA level and return to active daily living of the patients after discharge and in the early rehabilitation phase. Studies suggest that higher levels of PA before surgery can lead to better postoperative outcomes, including faster recovery and improved functional capacity [13, 14]. However, more evidence is needed to fully understand this relationship.

Low levels of PA have consequences such as increased all-cause mortality and chronic comorbidity [15]. This is also evident for knee OA patients [16]. On the contrary, regular PA at moderate or high intensity is associated with substantial health benefits [17]. Thus, evidence on improving PA with the perspective of a better post-knee replacement outcome is needed.

Only a few studies have objectively evaluated PA levels in OA patients, and thus, the influence of optimizing PA following knee replacement still needs to be answered. In a Dutch study, De Groot et al. (2007) observed that PA levels of patients with end-stage OA of the hip and knee were reduced (11%) compared to matched healthy controls [18]. Reduced PA was also found in Swedish patients scheduled for TKA compared to a healthy population [19]. In addition, it was shown that age and body mass index (BMI) were negatively associated with PA levels, indicating that specific interventions to improve PA, especially for heavier and older OA patients, are needed. Recently, a Danish study on fast-track TKA observed that PA levels were significantly reduced three weeks following TKA compared to preoperatively, calling for early stratified physiotherapeutic interventions [9]. The overall post-surgical improvement in physical activity for knee replacement patients has been questioned. Holsgaard-Larsen and Roos (2012) [18] found that PA levels remained low after surgery. Similarly, Kahn and Schwarzkopf (2016) [19] concluded that post-surgery activity levels were not significantly higher than pre-surgery levels. Furthermore, Hodges et al. (2018) [20] concluded that PA levels improved after TKA, but 12 months after surgery, about half of the patients did not meet the World Health Organization's recommendation for activity. This calls for improved and perhaps also individually stratified rehabilitation strategies in patients at risk for reduced postoperative PA and function [21].

### Health technology

Health technology, such as wearables and motivational feedback using gamification and nudging principles, are new features within health science. Gamification is traditionally defined as using game mechanics in non-gaming contexts to engage audiences [18] while nudging is a subtle and non-coercive method to influence decisions and behaviors for individual or societal benefit [22]. The use of smartphone apps and wearable devices in monitoring patients’ recovery are on the rise. Constantinescu et al. (2022) [23], in a systematic review, emphasized the potential of the technology to improve rehabilitation and increased self-engagement in TKA patients. However, the review concluded that more research is needed to fully understand the long-term effects and maximise the potential of these technologies. This highlights the need for further exploration into how motivational feedback, through nudging and gamification strategies, can be integrated to sustain physical activity and optimize health outcomes. Incorporating wearables and principles of motivational feedback such as gamification and nudging into health science could catalyze an individualized approach focused on increasing patient motivation for PA with expected improved physical functioning, faster and safer return to work, and increased quality of life. Thus, integrating individual rehabilitation goals into wearables may prioritize a more personalized approach and, thereby, potentially improve the effectiveness of rehabilitation following knee replacement surgery. The use of gamification and nudging has been shown to increase PA in overweight and obese adults [24]. Furthermore, a recent RCT study from Van der Walt et al. (2018) [25], on TKA and THA patients has shown that feedback, only on step counts per day, can play a crucial role in motivating patients to increase their daily PA.

SENS motion® (SENS Innovation ApS, Copenhagen, Denmark [26]) is a wireless medical accelerometer for collecting objective data on PA from large cohorts of patients. SENS motion® has developed a patient app with motivational feedback (Appendix 1, Fig. 3; App interface). The app has shown that hospitalized elderly patients with heart and lung diseases were motivated to be physically active for 51 min more each day [27]. The SENS system’s ability to monitor PA has been validated on knee-OA patients [28] and thus, the patient app with motivational feedback may provide a clinically relevant impact on patient self-mobilisation following discharge from knee replacement surgery.

## Objectives of the studies

This multi-center randomized controlled trial (RCT) nested in a prospective cohort aims to investigate whether PA following knee replacement surgery (TKA or mUKA) can be optimized using an activity monitoring device, including motivational feedback, compared with activity monitoring without feedback (care-as-usual). Furthermore, a prospective cohort will investigate the predictive value of PA level prior to knee replacement for the post-surgical length of stay, return to work, and quality of life.

### Hypotheses

#### RCT study

Using an activity monitoring device the first 12 weeks after discharge, including visual and motivational feedback on PA, shows a superior effect on increased PA (primary outcome), steps per day, minutes of PA, self-reported PA, self-reported function and pain, quality of life, global perceived effect on patient experience with knee problems, and return to work (secondary outcome measures) compared to no motivational feedback from the activity monitor, defined as 'care as usual', in knee replacement patients.

#### Prospective cohort

PA prior to knee replacement is a predictive measure for length of hospital stay, post-surgical function, quality of life, and return to work.

## Methods/Design

### Patient involvement

The current hypotheses have been discussed with our local patient panel, and it has raised various considerations about how the patient app should provide motivational feedback. These considerations include the choice between using a smartphone or a tablet, the delivery of feedback through notifications or on request, and whether the feedback should align with official PA guidelines or be customized to the individual's progress.

Subsequently, qualitative interviews were conducted with patients (*n* = 10) who had tested the app for one month with the aim of providing input for improvements of a prototype of the patient app. Feedback from these interviews suggested minor improvements that were implemented in an updated patient-specific app version before the inclusion of the first patient.

### Study design

**Fig. 1:**
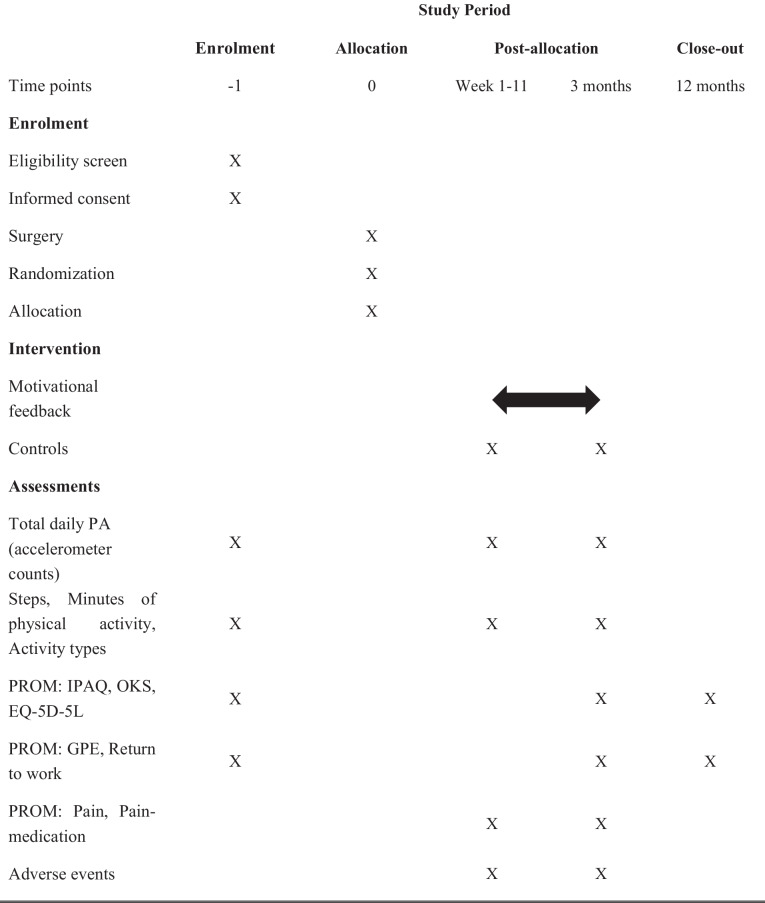
SPIRIT Figure. Template of content for the schedule of enrollment, interventions, and assessments. Abbreviations: PA: Physical activity; PROM: Patient-reported outcome measures; IPAQ: The International Physical Activity Questionnaire; OKS: Oxford Knee Score; EQ-5D-5L: Health-related quality of life; GPE: Global Perceived Effect

The RCT is a multicenter randomized (1:1) parallel-group superiority trial with a blinded statistical analysis toward group allocation (level of evidence: II) nested in a longitudinal prospective cohort study. The cohort study includes patients unwilling to participate in the RCT study. The study protocol adheres to the SPIRIT Statement (Standard Protocol Items: Recommendations for Interventional Trails) (see Additional file 1 for the SPIRIT Checklist and Fig. 1 for the SPIRIT Figure). The reporting of the RCT study will follow the CONSORT Statement (Consolidated Standards of Reporting Trials) [29], while the cohort study will be reported in accordance with the STROBE statement [30].

### Participants, randomization, and blinding

#### Participants and settings

Patients will be recruited from the Department of Orthopedics and Traumatology, Odense University Hospital, Denmark, and the Department of Orthopedic Surgery, Lillebaelt Hospital, Vejle, Denmark. Prior to inclusion, eligible patients (Table 1; Inclusion and exclusion criteria), will receive verbal and written information about the conditions of the trial and sign a standardized consent form. The longitudinally prospective cohort study includes patients unwilling to participate in the RCT study.

**Table 1:** Inclusion and exclusion criteria for patients in the study

Inclusion	Exclusion
Age: 40–85 years	Difficulty adhering to the study protocol
Patients scheduled for primary TKA/mUKA	Refusal of standard care
Understanding both verbal and written Danish language	Known- or newly diagnosed malignancy or palliative care
	Participation in an interventional clinical trial during the last 3 months potentially interacts with the aims of the current study
	Do not own a smartphone
	Undergone surgery using robot-assisted technology (CAS)

*Abbreviations: TKA* Total knee arthroplasty, *mUKA* Medial uni-compartmental knee arthroplasty

#### Randomization

Randomization is performed internet-based using REDCap Randomize, allocated 1:1. Randomization occurs after baseline measurements on the day of surgery. The randomization is performed in blocks of 2, 4, and 6. No stratifications are applied to the randomization, and the primary investigator is blinded regarding the permuted blocking strategy. A data manager, with no clinical involvement in the trial, prepares the randomization sequence. The allocation is concealed in a password-protected computer file that is only accessible to relevant personnel.

#### Blinding

The primary investigator (CDS) will be blinded to allocation and will not participate in the randomization of participants. The statistical analysis will only be performed on allocation codes; thus, the data analysts will be blinded concerning intervention allocation. Blinding of patients, surgeons, and nurses (healthcare providers) will not be possible due to the nature of the intervention.

### Sample size calculation and statistical procedures

To our knowledge, there are no anchor-based minimum clinically important differences (MCID) specifically for physical activity in knee patients. As a substitute, we use an MCID for steps per day, derived from chronic obstructive pulmonary disease research, corresponding to a change of approximately 17%–35% [31]. Applying a 17% threshold to total accelerometer counts per day, based on a previous study of TKA patients [9], yields an anticipated between-group difference in change score of 50,500 activity counts per day. This expected difference is further supported by findings from Van der Walt et al. (2018) [25], who demonstrated a 17% increase in daily step count in the feedback group compared to the non-feedback group after six months. To achieve 80% statistical power (β = 0.80) and detect statistically significant differences at a two-tailed α level of 0.05, assuming a standard deviation of 101,000 counts per day before and after the intervention [9] a sample size of 62 participants per group is estimated. To account for potential dropouts, we use a rate of 20% based upon evidence from a systematic review showing that dropout rates in RCTs on physical activity are within this percentage [32]. Consequently, we will include a sample of 150 patients in total. When appropriate, all outcome measures will be checked for Gaussian distribution using QQ-plots and parametric statistical and/or non-parametric analyses.

No a priori sample size calculation is made for the cohort study. However, based on a total sample of approximately 1,400 annual knee replacement procedures for OUH and Vejle hospitals, it is reasonable to expect an inclusion of 200 patients, which is acceptable for the current statistical analysis plan on predictive regression models [30].

The main comparative analyses between groups will be performed using an intention-to-treat analysis. Between-group mean differences and 95% confidence intervals will be estimated with a linear regression model. The patient’s baseline score is entered as a covariate and adjusted for potential baseline differences (age, sex, BMI). In addition to the intention-to-treat analysis, a per-protocol analysis will be conducted on patients adhering to the intervention (using the patient app for 5 out of 7 days in a week or > 70% of the available days.

### Intervention

Two weeks prior to surgery and 12 weeks following discharge, patients will be equipped with a discrete patch and built-in accelerometer (SENS motion®, Denmark, Copenhagen) attached to the thigh of the leg not undergoing surgery. The patch will be worn 24 h a day, and there will be no need to remove the patch before the end of the study (2 + 12 weeks). Besides raw accelerometer counts (primary outcome measure), the accelerometer also measures daily activity, daily steps, and type of activity. Prior to this protocol, a pilot study evaluated the validity of the SENS accelerometer on a similar patient group, showing that SENS motion® can measure PA and differentiate between different types of activities, such as lying down, sitting, standing, and standing work [33]. Before discharge patients randomized to the intervention group will receive a tablet with the app (*“SENS motion”*). To simplify usage, the tablet only contains the "SENS motion" app.

Patients randomized to the intervention group will be motivated to be more physically active through elements of gamification (e.g. achieving step goals) and nudging (supporting notifications). The sensor will measure the PA of the patient and accelerometer counts, irrespectively of the type of activity, will be converted into daily steps, which will be visible to the patients. Through the app, patients will be able to choose between two user interfaces. Interface 1 (Fig. 3B) allows patients to view predefined goals, including locations in a self-selected city whereas Interface 2 (Fig. 3A) provides graphical representations of their daily activity and their history during the period they have worn the accelerometer. The graphical representations include daily steps, physically active minutes, and type of activity. The motivational intervention is reported and described using “The template for intervention description and replication” (TIDieR) [34].

Data from SENS motion sensors are encrypted directly on the sensor itself before being sent to the server. The data is stored pseudonymized with a user ID. The server is located in Germany, and for security reasons, the exact location cannot be disclosed.

### Compliance

Both groups will have an app installed on a dedicated tablet (the intervention group) or their smartphone (the control group). For the control group, the app does not provide motivational feedback but is solely used to transfer data from the activity tracker to a central research database. To prevent data loss, weekly SMS messages will be sent to both groups with the message "Have you opened your SENS app this week?”. Furthermore, the researchers can verify proper attachment by following the skin temperature evaluated by the sensor via a web module. If the temperature fluctuates over an extended period, a project manager will call the patient to solve potential issues. A standard for adherence to app interventions has yet to be established. However, based on patient feedback, an adherence rate of 70%, meaning that the intervention group will use the app along with motivational feedback for 5 out of 7 weekdays, seems reasonable.

All patients will receive oral and written information on how to attach the sensor, use the app (without a tablet), and use the tablet with motivational feedback. Moreover, all patients will receive a link to a YouTube video presenting the project and providing instructions on how to use the equipment.

### Timing of assessments

**Fig. 2:**
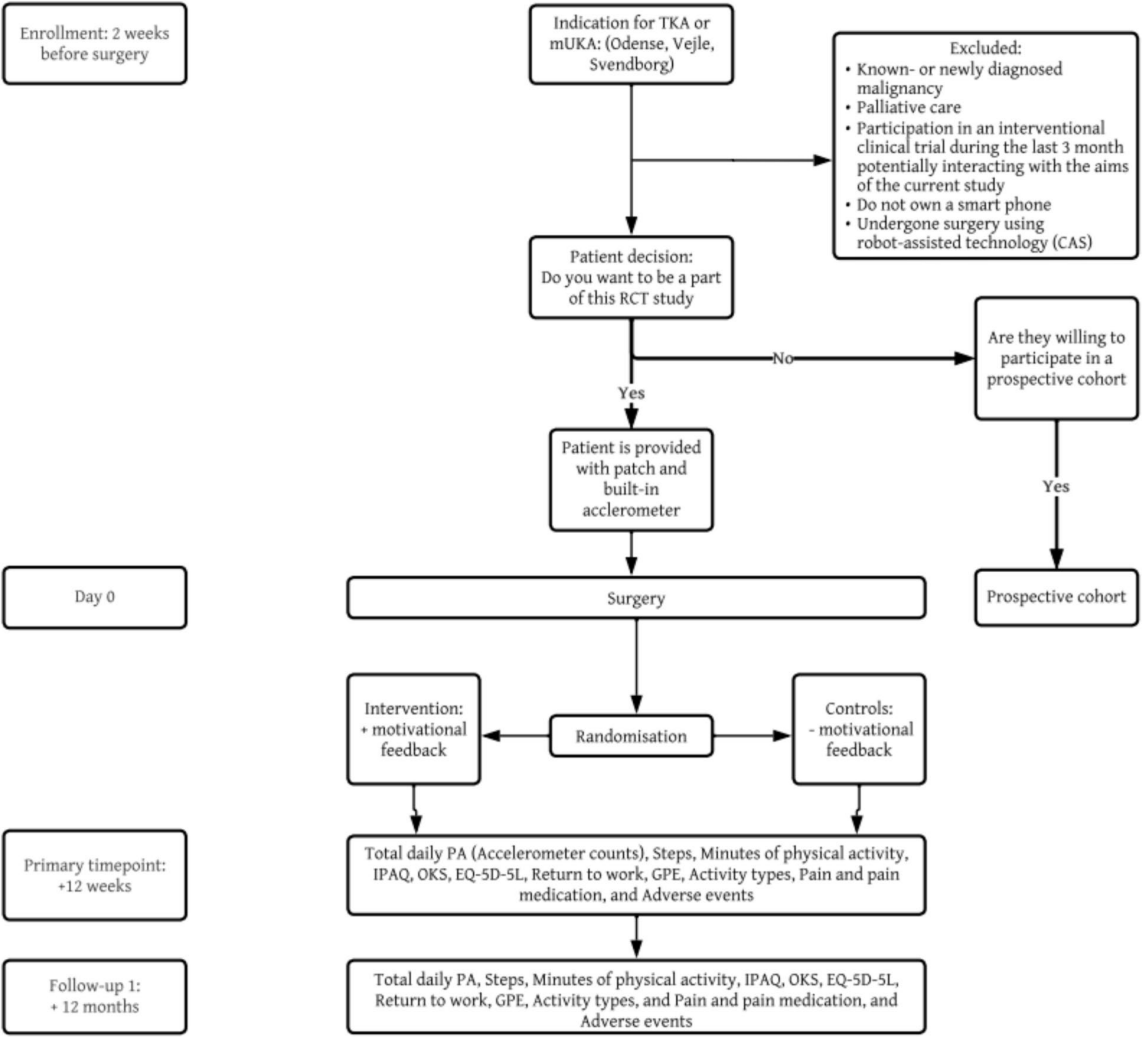
Flowchart of the study. Abbreviations: TKA: Total knee arthroplasty; mUKA: Medial uni-compartmental knee arthroplasty; PA: Physical activity; IPAQ: The International Physical Activity Questionnaire; OKS: Oxford Knee Score; EQ-5D-5L: Health-related quality of life; GPE: Global Perceived Effect

**Fig. 3:**
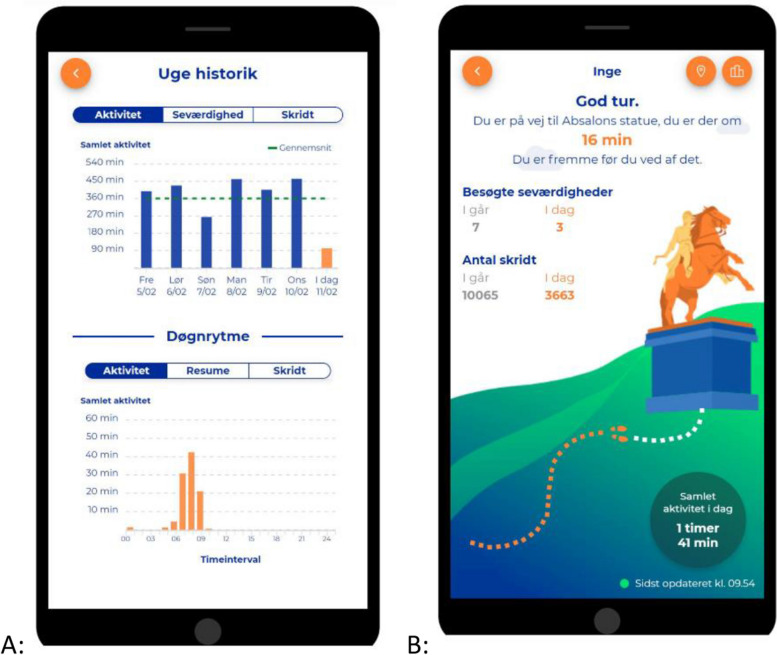
**A** Example of the graphical feedback screen (In Danish).  **B** Example of the visual feedback screen. Pictures: Healthcare – SENS Innovation ApS, used with consent

Assessment will be performed at baseline (two weeks prior to surgery and randomization), during the intervention (12 weeks post-surgery) (the primary endpoint), and a long-term follow-up on patient-reported outcomes will be performed at 12 months post-surgery (Fig. 2; Flowchart of the study and Table 2: Outcome measurements).

**Table 2:** Outcome measurement

	**Data Collection instrument**	**Collection time**			
		Baseline (2 weeks prior to surgery)	Post surgery (week 1–11)	Post surgery (3 months)	Post surgery (12 months)
**Primary Outcomes**					
Total daily PA (Accelerometer counts)	Accelerometer	X	X	X	
**Secondary outcomes**					
Steps	Accelerometer	X	X	X	
Minutes of physical activity	Accelerometer	X	X	X	
IPAQ	PROM	X		X	X
OKS	PROM	X		X	X
EQ-5D-5L	PROM	X		X	X
Return to work	PROM	X		X	X
GPE	PROM	X		X	X
**Tertiary outcomes**					
Activity types	Accelerometer	X	X	X	
Pain and pain medication	PROM, SMS-survey		X	X	
Adverse events	PROM		X	X	

Patient characteristics (Age, gender, BMI, work type and surgery leg) will be obtained at baseline

*Abbreviations: PA* Physical activit, *IPAQ* The International Physical Activity Questionnaire, *PROM* Patient-reported outcome measures, *OKS* Oxford Knee Score, *EQ-5D-5L* Health-related quality of life, *GPE* Global Perceived Effect

## Outcome measures

### Patient characteristics

At baseline prior to randomization, height and weight will be measured, and gender, age, work type, and surgery leg will be recorded. After randomization (post-surgery), the type of knee arthroplasty (TKA/mUKA) and length of hospital stay will be obtained from patient journals.

### Primary outcome measure

PA will be evaluated by SENS motion® as described above. The primary outcome measure is the between-group change score of total daily PA (accelerometer counts) from baseline to 12 weeks following surgery. Accelerometer counts per day is a cumulative and validated variable based on raw accelerometer data in 3 planes and is a proxy for total daily PA [35].

### Secondary outcome measures

#### Steps

Steps per day are measured with SENS motion® [28, 33].

#### Minutes of physical activity

Minutes of physical activity per day measured with SENS motion®.

#### Self-reported physical activity

Patient-reported PA will be measured by The International Physical Activity Questionnaire Short Form (IPAQ-SF), which is a 7-item questionnaire consisting of open-ended questions surrounding the patient's last seven days' recall of PA [36, 37]. The short and long version of the IPAQ was translated into Danish, and the long version was validated in 2014 [38].

#### The Oxford Knee Score

The Oxford Knee Score (OKS) is a validated 12-item patient-reported questionnaire designed and developed to assess function and pain following TKA [39]. It was translated and validated into Danish in 2009 [40]. The questionnaire generates scores ranging from 0–48, with a score of 48 representing the best outcome post-TKA. Each item response is graded between 0–4.

#### General Health

The EQ-5D-5L is a validated patient-reported questionnaire assessing health-related quality of life and consists of the following dimensions: mobility, self-care, usual activities, pain/discomfort, and anxiety/depression. Each dimension uses a 5-point Likert scale [41]. The EQ-5D-5L has been validated in knee OA patients referred for knee arthroplasty surgery [42].

#### Return to work

At 12 weeks and 12 months follow up, all patients will receive tree questions about changes in work status (retried/sick leave/job-seeking), work type (non-physically demanding/physically demanding), and working time (full-/part-time).

#### Global Perceived Effect (GPE)

At 12 weeks and 12 months follow-up, two GPE questions will be sent to the patient. A 7-point Likert scale using the following answers “Worse," "Slightly worse," “Very slight worse", "The same, "Minimal improvement," "Slightly better," and "Better” will be used to answer the following question: “How do you experience your knee problems now, compared to before the surgery?”.

### Tertiary outcome measures

#### Activity types

Activity types evaluated by SENS motion®, i.e. time spent sleeping/resting, standing, walking, and cycling, will be considered.

#### Pain and pain medication

The patients receive weekly text messages (SMS) with a question about their knee pain the previous week. They will be asked to rate their pain on an NRS scale from 0–10, where 0 corresponds to no pain and 10 corresponds to the worst imaginable pain. Furthermore, patients will receive an SMS with a question about their use of pain medication in the last week. The SMS has four options: i) No use of pain medication the past week, ii) use of non-prescription medicine (e.g., Panodil, Pamol, Ibumetin) at least once during the past week, iii) use of strong painkillers (e.g., Morphine, Tramadol) at least once the past week, and iv) use of both non-prescription medicine and strong painkillers at least once the past week.

### Adverse events

Reporting of adverse events will be elicited through self-reported questionnaires 30 and 90 days after the operation. All events will be coded according to the Medical Dictionary for Regulatory Activities, as currently required by all regulatory authorities, including the US Food and Drug Administration and the European Agency for the Evaluation of Medicinal Products. Additionally, a project staff member will examine the patient's medical record for more severe adverse events 7 and 90 days after TKA/mUKA (e.g. deep vein thrombosis (DVT), lung emboli (LE), death, and re-operation). The motivational intervention for increased PA is non-invasive, and only minor discomfort associated with the procedure is anticipated. In cases of unacceptable pain (NRS > 5) for consecutive days, the project manager can be directly contacted. Patients are informed to follow standard procedures for PA after knee replacement in which it is specified that patients should be aware of signs such as fatigue and pain, which indicate the need to take a break from exercise.

## Discussion

This randomized controlled superiority multi-center trial will evaluate the effect of activity monitoring and motivational feedback following knee arthroplasty surgery. The RCT study is expected to provide high-level evidence of the potential clinical and functional benefits of activity monitoring and motivational feedback after a TKA/mUKA. This trial is a multicenter RCT, which results in increased external validity and generalizability of the results. While the literature demonstrates consistent results on decreased levels of post-surgical PA, there is limited understanding of how to improve post-surgical PA levels and the return to active daily living following knee replacement surgery. Existing studies indicate that many knee patients do not achieve significant improvements after surgery and do not meet World Health Organization recommendations for activity [20]. Consequently, there is a need for interventions that help patients enhance post-surgical physical activity.

### Outcome variables

The primary outcome variable, the between-group change score of total daily PA (accelerometer counts) after 12 weeks, is chosen to examine if short-term PA can be increased with motivational feedback. Furthermore, patient-reported outcome variables are evaluated at 12-month follow-up to evaluate potential long-term effects of the intervention on knee function and quality of life. In addition to the objective PA measurements, patients will respond to a questionnaire regarding their subjective PA. Both approaches are combined to provide a more nuanced understanding of the patients’ PA.

The 12-week follow-up was chosen to identify possible improvements at the end of the acute rehabilitation period, where patients are expected to have returned to an active lifestyle and/or have ended their sick leave. 12 weeks is also a reasonably long timeframe for clinical improvements to occur in patients who have undergone knee-arthroplasty surgery, yet short enough to assume that patients would be able to recall their baseline condition. The 12-month follow-up was selected to capture any potential changes beyond the short-term rehabilitation as an expression of a long-term effect.

### Study design

Prior to this RCT, a qualitative study was conducted including 10 TKA/mUKA-patients. Integrating qualitative research before the RCT strengthens the study design by understanding our population and their perspectives. Furthermore, the qualitative study helped optimize the app for the population and their specific needs. This approach facilitates a more comprehensive and well-informed methodology for conducting rigorous and impactful research.

Only a few exclusion criteria will be employed to improve external validity and generalizability. However, the study may potentially be affected by selection bias. An exclusion criterion of “do not own a smartphone” will be used since the use of a smartphone is necessary for the intervention. Furthermore, the trial is based on patients volunteering for a physical intervention, which can also be a cause of selection bias. The accelerometer used in this trial has a maximum memory capacity of 7–14 days. To prevent data loss resulting from a full memory, it is required to establish a weekly data transfer connection to a smartphone using the dedicated application. Therefore, we decided to send a weekly SMS reminder to patients in the intervention and control groups. This approach may introduce attention bias. Moreover, all patients, whether in the intervention or control group, will be aware of wearing the accelerometer, which also may introduce attention bias. However, using their own smartphones to transfer data should minimize the risk of bias in the control group, resulting in more accurate PA levels for comparison.

Public access to the current protocol paper and prior registration on ClinicalTrials.gov will guarantee transparency regarding the implemented methods and definitions of outcome measures.

### Perspective

If proven effective, the use of gamification tools and accelerometers introduced in this study can potentially enhance the current rehabilitation process for patients undergoing knee replacement. Furthermore, the use of gamification tools and accelerometers is extensive, and if proven effective, findings might be extrapolated to other conditions where a rehabilitation process plays a crucial role in the patient's return to an active life.

## Supplementary Information

Additional file 1: SPIRIT Checklist. Spirit 2013 Checklist: Recommended items to address in clinical trial protocol and related documents (DOC 71,5 KB).

Additional file 2: “The template for intervention description and replication” (TiDieR) Checklist (DOC: 65,0 KB).

## Abbreviations

OA: Osteoarthritis
PA: Physical Activity
TKA: Total knee arthroplasty
mUKA: Medial uni-compartmental knee arthroplasty
BMI: Body Mass Index
RCT: Randomized controlled trial
INT: Intervention group
CON: Control group
IPAQ: The International Physical Activity Questionnaires
OKS: Oxford Knee Score
EQ-5D-5L: Health-related quality of life
GPE: Global perceived effect
ADL: Activities of Daily Living
QOL: Quality of life
NRS: Numeric Rating Scale
DVT: Deep vein thrombosis
LE: Lung emboli
OUH: Odense University Hospital
SDU: University of Southern Denmark
