# Supplementary material for: Impact of motivational feedback on levels of physical activity and quality of life by activity monitoring following knee arthroplasty surgery—protocol for a randomized controlled trial nested in a prospective cohort (Knee-Activity)
Source: BMC Musculoskelet Disord. 2024 Oct 2;25:778. doi: 10.1186/s12891-024-07878-0 (PMC11448174; doi:10.1186/s12891-024-07878-0)
Supplement: Supplementary file 1 — Additional file 1: SPIRIT Checklist. Spirit 2013 Checklist: Recommended items to address in clinical trial protocol and related documents (DOC 71,5 KB). [file 12891_2024_7878_MOESM1_ESM.docx]

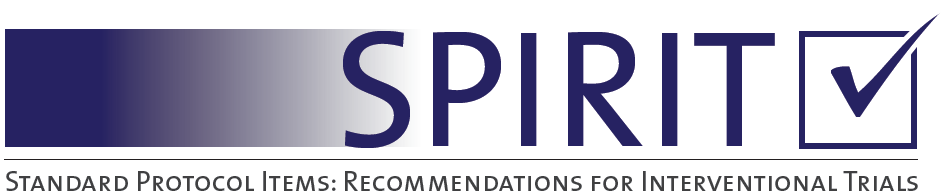


SPIRIT 2013 Checklist: Recommended items to address in a clinical trial protocol and related documents*

| Section/item | ItemNo | Description |
| --- | --- | --- |
| **Administrative information** | | |
| Title | 1 | **Descriptive title identifying the study design, population, interventions, and, if applicable, trial acronym**  Impact of *motivational feedback* on levels of physical activity and quality of life by activity tracking *following knee arthroplasty surgery* - protocol for *a randomized controlled trial nested in a prospective cohort* (*Knee-Activity*). |
| Trial registration | 2a | **Trial identifier and registry name. If not yet registered, name of intended registry**  ID: NCT06005623. Registered on 2023-08-22. |
|  | 2b | **All items from the World Health Organization Trial Registration Data Set** |
| Protocol version | 3 | **Date and version identifier**  Under “Trial registration”  Registered on 2023-08-22.   - Protocol version 4 |
| Funding | 4 | **Sources and types of financial, material, and other support**  Financial:   - The Faculty of Health Sciences, - The Region of Sputhern Denmark, - Department of Orthopedic Surgery and Traumatology, - GigtForeningen.   For further information, see *“Funding”* (Page: 18, Line: 376-379). |
| Roles and responsibilities | 5a | **Names, affiliations, and roles of protocol contributors**  This information can be found below *“Authors’ contributions”* (Page: 18, Line: 380-385). |
|  | 5b | **Name and contact information for the trial sponsor**  This information can be found below *“Authors’ contributions”* (Page: 18, Line: 380-385). and on the title page. |
|  | 5c | **Role of study sponsor and funders, if any, in study design; collection, management, analysis, and interpretation of data; writing of the report; and the decision to submit the report for publication, including whether they will have ultimate authority over any of these activities**  This information can be found below *“Authors’ contributions”* (Page: 18, Line: 380-385) and below *“Competing interests”* (Page: 18, line 372-375). |
|  | **5d** | **Composition, roles, and responsibilities of the coordinating centre, steering committee, endpoint adjudication committee, data management team, and other individuals or groups overseeing the trial, if applicable (see Item 21a for data monitoring committee)** |
| Introduction |  |  |
| Background and rationale | 6a | **Description of research question and justification for undertaking the trial, including summary of relevant studies (published and unpublished) examining benefits and harms for each intervention**  The background section contains the relevant information about the number of individuals who undergo TKA/mUKA and the percentage of those who do not return to the active lifestyle they desire. Additionally, the background section includes information about motivating feedback, gamification, and nudging, as well as the research conducted in this area. In the background section, you can also find information about the equipment we use in the study and the research conducted on this specific accelerometer. |
|  | 6b | **Explanation for choice of comparators**  The control group used in this randomized controlled trial (RCT) is similar to the intervention group. There is no difference in the groups' course of action concerning pre-examination, surgery, or follow-up. Inclusion and exclusion criteria have been established (see Table 1), where point 5, which requires the patient to have a smartphone, is expected to create the most significant selection bias. However, a smartphone is necessary for the intervention to function. |
| Objectives | 7 | **Specific objectives or hypotheses**  This information can be found below *“Hypotheses”* (Page: 6-7, Line: 123-132). |
| Trial design | 8 | **Description of trial design including type of trial (eg, parallel group, crossover, factorial, single group), allocation ratio, and framework (eg, superiority, equivalence, noninferiority, exploratory)**  The RCT is a multicenter randomized (1:1) parallel-group superiority trial, nested in a longitudinal prospective cohort study, with blinded statistical analysis towards group allocation (level of evidence: II).  For further information, see *“Study design”* (Page: 7-8, Line: 144-151). |
| Methods: Participants, interventions, and outcomes | | |
| Study setting | 9 | **Description of study settings (eg, community clinic, academic hospital) and list of countries where data will be collected. Reference to where list of study sites can be obtained**  Patient recruitment takes place at the following sites:   - Odense University Hospital, Department of Orthopedic Surgery, Denmark - Svendborg Hospital, Department of Orthropedic Surgery, Denmark - Lillebaelt Hospital, Department of Orthopedic Surgery, Vejle, Denmark   For further information, see *“Participants and settings”* (Page: 8, Line: 153-158). |
| Eligibility criteria | 10 | **Inclusion and exclusion criteria for participants. If applicable, eligibility criteria for study centres and individuals who will perform the interventions (eg, surgeons, psychotherapists)**  This information can be found in Table 1. |
| Interventions | 11a | **Interventions for each group with sufficient detail to allow replication, including how and when they will be administered**  This information can be found below “*Intervention”* (Page: 10, line: 191-213) and in figure 2. |
|  | 11b | **Criteria for discontinuing or modifying allocated interventions for a given trial participant (eg, drug dose change in response to harms, participant request, or improving/worsening disease)**  Patients are informed to follow standard procedures for PA after knee replacement in which it is specified that patients should be aware of signs such as fatigue and pain, which indicate the need to take a break from exercise. In cases of unacceptable pain (NRS > 5) for consecutive days, the project manager can be directly contacted.  For further information, see *“Adverse events”* (Page: 14, Line: 286-297). |
|  | 11c | **Strategies to improve adherence to intervention protocols, and any procedures for monitoring adherence (eg, drug tablet return, laboratory tests)**  Both groups will have an app installed on a dedicated tablet (the intervention group) or their smartphone (the control group) to secure data transfer. To prevent data loss, weekly SMS messages will be sent to both groups with the message "Have you opened your SENS app this week?”  For further information, see *“Compliance”* (Page: 11, Line: 214-227). |
|  | 11d | **Relevant concomitant care and interventions that are permitted or prohibited during the trial**  Not applicable – The patients should live as normally as possible, and there are no restrictions associated with the project |
| Outcomes | 12 | **Primary, secondary, and other outcomes, including the specific measurement variable (eg, systolic blood pressure), analysis metric (eg, change from baseline, final value, time to event), method of aggregation (eg, median, proportion), and time point for each outcome. Explanation of the clinical relevance of chosen efficacy and harm outcomes is strongly recommended**  Patient characteristics  Primary outcome measure   - The primary outcome measure is the between-group change score of total daily PA (accelerometer counts) from baseline to 12 weeks following surgery   Secondary outcome measures   - Steps - Minutes of physical activity - Self-reported physical activity - The Oxford Knee Score - General Health - Return to work - Global Perceived Effect (GPE)   Tertiary outcome measures   - Activity types - Pain and pain medication   Adverse Events  Further information about each outcome can be found below “*Outcome measures*” (Page: 12-14, Line: 234-297) and in Figure 1. |
| Participant timeline | 13 | **Time schedule of enrolment, interventions (including any run-ins and washouts), assessments, and visits for participants. A schematic diagram is highly recommended (see Figure)**  This information can be found in Figure 2: Flowchart of the study |
| Sample size | 14 | **Estimated number of participants needed to achieve study objectives and how it was determined, including clinical and statistical assumptions supporting any sample size calculations**  The sample size calculation can be found under “Sample size” (Page: 9, Line: 171-190) |
| Recruitment | 15 | **Strategies for achieving adequate participant enrolment to reach target sample size**  Based upon a total sample of approximately 1,400 annual knee replacement procedures for OUH and Vejle hospitals it is reasonable to expect an inclusion of 200 patients in the cohort and 150 in the RCT. |
| **Methods: Assignment of interventions (for controlled trials)** | | |
| Allocation: |  |  |
| Sequence generation | 16a | **Method of generating the allocation sequence (eg, computer-generated random numbers), and list of any factors for stratification. To reduce predictability of a random sequence, details of any planned restriction (eg, blocking) should be provided in a separate document that is unavailable to those who enrol participants or assign interventions**  Randomization is performed internet-based using REDCap Randomize, allocated 1:1. Randomization occurs after baseline measurements on the day of surgery. |
| Allocation concealment mechanism | 16b | **Mechanism of implementing the allocation sequence (e.g., central telephone; sequentially numbered, opaque, sealed envelopes), describing any steps to conceal the sequence until interventions are assigned**  The allocation is concealed in a password-protected computer file accessible only to relevant personnel. |
| Implementation | 16c | **Who will generate the allocation sequence, who will enrol participants, and who will assign participants to interventions**  A data manager with no clinical involvement in the trial prepares the randomization sequence |
| Blinding (masking) | 17a | **Who will be blinded after assignment to interventions (e.g., trial participants, care providers, outcome assessors, data analysts), and how**  The primary investigator will be blinded to allocation and will not participate in randomizing participants. The statistical analysis will be performed on allocation codes only and thus, the data analysts will be blinded concerning intervention allocation.  Blinding of patients, surgeons, and nurses (healthcare providers) will not be possible due to the nature of the intervention. |
|  | 17b | **If blinded, circumstances under which unblinding is permissible and procedure for revealing a participant’s allocated intervention during the trial** |
| **Methods: Data collection, management, and analysis** | | |
| Data collection methods | 18a | **Plans for assessment and collection of the outcome, baseline, and other trial data, including any related processes to promote data quality (e.g., duplicate measurements, training of assessors) and a description of study instruments (e.g., questionnaires, laboratory tests) along with their reliability and validity, if known. Reference to where data collection forms can be found, if not in the protocol**  Accelerometer:   - Total daily PA (accelerometer counts): Baseline, Follow-up 1, - Steps: Baseline, Follow-up 1, - Minutes of physical activity: Baseline, Follow-up 1, - Activity types: Baseline, Follow-up 1,   PROM   - Self-reported physical activity: Baseline, Follow-up 1, Follow up 2 - The Oxford Knee Score: Baseline, Follow-up 1, Follow up 2 - General Health: Baseline, Follow-up 1, Follow up 2 - Return to work: Follow-up 1, Follow up 2 - Global Perceived Effect (GPE): Follow-up 1, Follow up 2 - Adverse Events: Follow-up 1   SMS-surveys:   - Pain and pain medication: Follow-up 1   Information about each outcome can be found below “*Outcome measures*” (Page: 12-14, Line: 234-297) and in Figure 1. |
|  | 18b | **Plans to promote participant retention and complete follow-up, including a list of any outcome data to be collected for participants who discontinue or deviate from intervention protocols**  The patients will receive weekly SMS messages throughout the intervention period. Additionally, it is possible to contact the patients if the equipment is incorrectly positioned or becomes detached. Patients will receive one reminder about the questionnaires related to the trial. If patients do not respond to this reminder, it is possible to make phone contact with them. |
| Data management | 19 | **Plans for data entry, coding, security, and storage, including any related processes to promote data quality (e.g., double data entry; range checks for data values). Reference to where details of data management procedures can be found, if not in the protocol**  This information can be found below: *“Participants, randomization, and blinding”* (Page: 8-9), Line: 152-190 |
| Statistical methods | 20a | **Statistical methods for analysing primary and secondary outcomes. Reference to where other details of the statistical analysis plan can be found, if not in the protocol**  This information can be found below “*Sample size calculation and statistical procedures”* (Page: 9, Line: 171-190). |
|  | 20b | **Methods for any additional analyses (e.g., subgroup and adjusted analyses)**  This information can be found below *“Sample size calculation and statistical procedures”* (Page: 9, line: 171-190). |
|  | 20c | **Definition of analysis population relating to protocol non-adherence (e.g., as randomised analysis) and any statistical methods to handle missing data (e.g., multiple imputations)**  This information can be found below *“Sample size calculation and statistical procedures”* (Page: 9, line: 171-190). |
| **Methods: Monitoring** | | |
| Data monitoring | 21a | **Composition of data monitoring committee (DMC); summary of its role and reporting structure; statement of whether it is independent of the sponsor and competing interests; and reference to where further details about its charter can be found, if not in the protocol. Alternatively, an explanation of why a DMC is not needed**  A Data Monitoring Committee (DMC) has not been established because all treatments associated with the project are approved. Additionally, the equipment used is CE-marked for medical trials. The intervention is low-risk, and there are no expected severe adverse effects in connection with the project. |
|  | 21b | **Description of any interim analyses and stopping guidelines, including who will have access to these interim results and make the final decision to terminate the trial**  A data processing agreement has been made with Sens Motion ApS, where the following supervision takes place: The data processor must annually, at no cost, respond to and complete a questionnaire prepared by the data controller. Additionally, the data controller or a representative of the data controller has the right to ask the data processor additional questions at any time |
| Harms | 22 | **Plans for collecting, assessing, reporting, and managing solicited and spontaneously reported adverse events and other unintended effects of trial interventions or trial conduct**  Reporting of adverse events will be elicited through self-reported questionnaires 30 and 90 days after the operation. Additionally, a project staff member will examine the patient's medical record for more severe adverse events 7 and 90 days after TKA/mUKA (e.g. deep vein thrombosis (DVT), lung emboli (LE), death, and re-operation).  Further information about each outcome can be found below “*Adverse events*” (Page: 14, Line: 286-297). |
| Auditing | 23 | **Frequency and procedures for auditing trial conduct, if any, and whether the process will be independent of investigators and the sponsor**  Not applicable |
| Ethics and dissemination | | |
| Research ethics approval | 24 | **Plans for seeking research ethics committee/institutional review board (REC/IRB) approval**  The Regional Committee for Medical Research Ethics, Project ID: S-20222000-171 |
| Protocol amendments | 25 | **Plans for communicating necessary protocol modifications (e.g., changes to eligibility criteria, outcomes, analyses) to relevant parties (e.g., investigators, REC/IRBs, trial participants, trial registries, journals, regulators)**  The Danish Medicines Agency has declared no need for further permission to carry out the study. |
| Consent or assent | 26a | **Who will obtain informed consent or assent from potential trial participants or authorised surrogates, and how (see Item 32)**  Eligible patients will receive verbal and written information about the conditions of the trial and sign a standardized consent form. |
|  | 26b | **Additional consent provisions for collection and use of participant data and biological specimens in ancillary studies, if applicable**  Not applicable |
| Confidentiality | 27 | **How personal information about potential and enrolled participants will be collected, shared, and maintained to protect confidentiality before, during, and after the trial**  Data from SENS motion sensors are encrypted directly on the sensor before being sent to the server. The data is stored pseudonymised with a user ID. The server is located in Germany, and for security reasons, the exact location cannot be disclosed.  The dataset used and/or analysed during the current study will be housed on the project’s Sharpoint website, and all datasets will be password-protected. To ensure confidentiality, data dispersed to project team members will be blinded to any identifying participant information. |
| Declaration of interests | 28 | **Financial and other competing interests for principal investigators for the overall trial and each study site**  This information can be found below “*Competing interest*” (Page: 18, line 372-375). |
| Access to data | 29 | **Statement of who will have access to the final trial dataset and disclosure of contractual agreements that limit such access for investigators**  The datasets used and/or analysed during the current study are available from the corresponding author upon reasonable request. |
| Ancillary and post-trial care | 30 | **Provisions, if any, for ancillary and post-trial care and for compensation to those who suffer harm from trial participation**  Not applicable |
| Dissemination policy | 31a | **Plans for investigators and sponsor to communicate trial results to participants, healthcare professionals, the public, and other relevant groups (e.g., via publication, reporting in results databases, or other data-sharing arrangements), including any publication restrictions**  The study will be submitted for publication regardless of negative, positive, or inconclusive results. |
|  | 31b | **Authorship eligibility guidelines and any intended use of professional writers**  The four ICJME criteria has been followed |
|  | 31c | **Plans, if any, for granting public access to the complete protocol, participant-level dataset, and statistical code**  The datasets used and/or analyzed during the current study are available from the corresponding author upon reasonable request. |
| Appendices |  |  |
| Informed consent materials | 32 | **Model consent form and other related documentation given to participants and authorised surrogates**  Before inclusion, eligible patients (Table 1; Inclusion and exclusion criteria) will receive verbal and written information about the conditions of the trial and sign a standardized consent form. |
| Biological specimens | 33 | **Plans for collection, laboratory evaluation, and storage of biological specimens for genetic or molecular analysis in the current trial and future use in ancillary studies, if applicable**  Not applicable. |

*It is strongly recommended that this checklist be read in conjunction with the SPIRIT 2013 Explanation & Elaboration for necessary clarification on the items. Amendments to the protocol should be tracked and dated. The SPIRIT Group copyrights the SPIRIT checklist under the Creative Commons “[Attribution-NonCommercial-NoDerivs 3.0 Unported](http://www.creativecommons.org/licenses/by-nc-nd/3.0/)” license.
