# Supplementary material for: Impact of motivational feedback on levels of physical activity and quality of life by activity monitoring following knee arthroplasty surgery—protocol for a randomized controlled trial nested in a prospective cohort (Knee-Activity)
Source: BMC Musculoskelet Disord. 2024 Oct 2;25:778. doi: 10.1186/s12891-024-07878-0 (PMC11448174; doi:10.1186/s12891-024-07878-0)
Supplement: Supplementary file 2 — Additional file 2: “The template for intervention description and replication” (TiDieR) Checklist (DOC: 65,0 KB). [file 12891_2024_7878_MOESM2_ESM.docx]

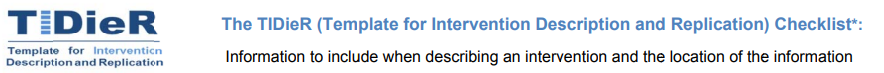
**Brief Name**

1. Knee-Activity: Activity tracking and motivational feedback following knee replacement surgery.

**WHY**

1. Despite successful elective surgical procedures, patients following total knee arthroplasty (TKA) and medial uni-compartmental knee arthroplasty (mUKA) still demonstrate decreased function and physical activity (PA), earlier retirement, less income, increased health costs, and home care compared to matched controls. Health technology, such as wearables and motivational feed-back using gamification and nudging principles, are new features within health science. Thus, wearables contain a clinically relevant potential to increase patient self-mobilisation following discharge from TKA and UKA.

**WHAT**

1. **Materials:** The patients are equipped with an activity tracker with a built-in accelerometer (SENS Motion, Denmark, Copenhagen). This accelerometer is attached to the thigh of the leg not undergoing surgery. Patients randomised to the intervention group will be provided with a ‘Lenovo smart TAB M8” (referred to as “Tablet” in the rest of this text). Data from the accelerometer is converted into a biomechanical interpretation of PA. These interpretations can be displayed in the web system as graphs, summarised on a daily basis, and converted into steps.

After the patient's surgery, the nursing staff in the department will create the patient's profile in the app and briefly introduce its functions. The patients will be provided with informational writing materials about the tablet. An introductory video about the tablet's platform has also been created, which is available to the patients.

1. **Procedures:** Firstly, the app provides graphical representations with numerical values representing the overall activity (in minutes), the type of activity (minutes spent lying down (bedridden), sitting, standing, and walking), and steps. The display showed the current day's activities and the past 6 days. Secondly, as a gamification feature, the patient will be taken on a visual tour of a city of their choosing, passing by various attractions. The tour will be reset every day. The display showed the current day's attractions together with the past 6 days.

**WHO PROVIDED**

- The accelerometer and the "Sens Motion" app combination will hopefully incentivise participants to enhance their physical activity levels by incorporating gamification and nudging elements. The sensor will assess the patient's PA and the resulting accelerometer data will be converted into daily step counts, which will be presented to the participants. Within the app, patients will have the option to select from two tabs. Tab 1 will enable patients to access predefined goals, all pertaining to locations within a city of their choosing. Tab 2 will grant patients access to visual representations of their daily activity and a historical record of their activity while wearing the accelerometer. These visual representations encompass daily step counts, minutes of physical activity, and the specific types of activities performed.

**HOW**

- The accelerometer should be worn continuously for 24 hours a day, and there is no need to remove it before the completion of the 14 weeks. Patients assigned to the intervention group will be advised to keep the tablet on their kitchen or dining table, ensuring it remains visible.

**WHERE**

1. The accelerometer will monitor the patient's daily activities, making the intervention applicable within the patients' homes.

**WHEN and HOW MUCH**

1. During the 12 weeks following their discharge after knee replacement surgery, patients will receive an accelerometer and tablet.

**TAILORING**

1. Patients within the intervention group can personally select one of six cities, resulting in potential variations in the attractions available to them. Additionally, the platform introduced to the intervention group will remain consistent for all patients.

**MODIFICATIONS**

1. After intervention

**HOW WELL**

1. Planned:

- To prevent data loss, weekly SMS messages will be sent to intervention and control groups with the message "Have you opened your SENS app this week?"
- Furthermore, the researchers can verify the proper attachment of the sensor via a web module. The sensor measures skin temperature, and if the temperature fluctuates over an extended period, a project manager will call the patient to solve potential issues.

1. Actual:

- After intervention
